# Smart metasurfaces enable wireless detection of hazardous liquids

**DOI:** 10.1093/nsr/nwaf319

**Published:** 2025-08-06

**Authors:** Zihao Dai, Vincenzo Galdi

**Affiliations:** Fields & Waves Lab, Department of Engineering, University of Sannio, Italy; School of Electronic Science and Engineering, University of Electronic Science and Technology of China, China; Fields & Waves Lab, Department of Engineering, University of Sannio, Italy

## Abstract

A wireless sensing platform combining smart metasurfaces, orthogonal frequency division multiplexing, and artificial intelligence achieves real-time, non-invasive classification of hazardous liquids in sealed containers with high accuracy and robustness.

Detecting hazardous liquids in sealed containers at airports, public venues or customs checkpoints remains a persistent challenge. Conventional techniques such as optical spectroscopy, chemical sensors and radio-frequency (RF) systems often involve direct contact, require precise calibration or depend on controlled environments. These limitations make real-time, non-invasive screening difficult in practical settings. In response to this challenge, researchers at Xidian University have developed a promising new approach that combines programmable metasurfaces, orthogonal frequency division multiplexing (OFDM) and artificial intelligence (AI) [1]. This integrated system may enable fast, contactless identification of multiple liquids with high accuracy and robust performance in real-world conditions.

At the core of the system is a space–time coding (STC) programmable metasurface. This structure consists of subwavelength unit cells that can be dynamically reconfigured in both space and time, enabling real-time beam steering and frequency harmonic generation [2]. Although STC metasurfaces have been widely explored in reconfigurable wireless environments and dynamic beamforming [3], their role in sensing has largely been limited to direction-of-arrival estimation [4], target detection and vital-sign monitoring [5]. This work demonstrates how they can be applied to material recognition through dielectric spectral signatures.

Figure 1 shows the system schematic. A horn antenna directs a signal onto the metasurface, which transforms the wavefront into multiple high-order harmonic beams. Each beam probes a separate region containing a liquid sample. Scattered signals are collected by a monopole antenna, digitized by a software-defined radio (universal software radio peripheral, USRP) and processed by a control computer. The metasurface is driven by a field-programmable gate array (FPGA) that enables fast and flexible spatio-temporal modulation.

**Figure 1.**
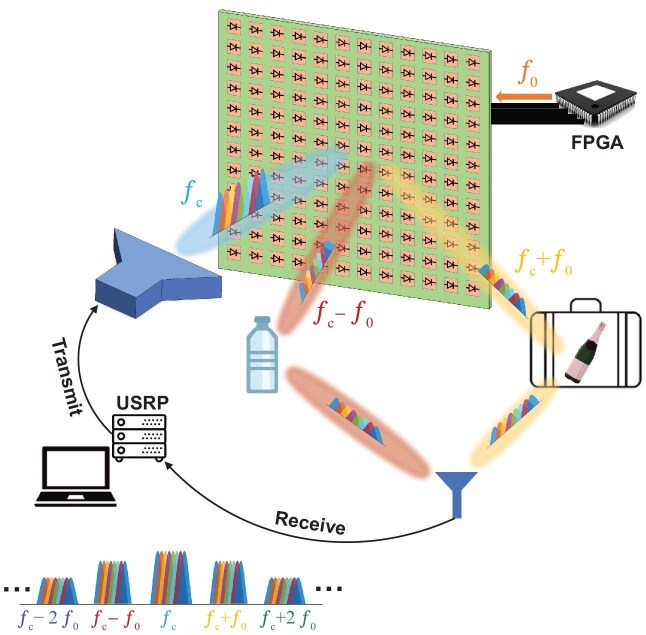
Conceptual illustration of the wireless liquid detection system. A horn antenna transmits OFDM signals toward an STC metasurface. The metasurface, driven by an FPGA, dynamically modulates incident waves into multiple harmonic beams, each probing a different liquid sample. Scattered signals are collected by a monopole antenna, digitized by a USRP, and processed by AI algorithms on a control computer for real-time liquid classification.

The system operates at 5.8 GHz and uses OFDM, a multicarrier modulation scheme widely adopted in wireless communications for its spectral efficiency and robustness against multipath fading. In this sensing configuration, OFDM provides frequency diversity across its subcarriers, allowing the system to capture subtle differences in scattering behavior caused by variations in dielectric properties. The harmonic beams generated by the metasurface interact with the liquids, and their scattering responses are encoded in the channel state information.

The amplitude of the OFDM subcarriers is used as a robust feature vector for classification. Three AI models were evaluated: support vector machines (SVMs), decision trees (DTs) and convolutional neural networks (CNNs) [6]. CNN and DT models consistently achieved the highest performance, exceeding 99% classification accuracy in single-liquid and multi-liquid scenarios. SVMs performed slightly less well, with accuracy between 82% and 90%, particularly under more complex configurations.

The system was validated under varying container volumes, materials and spatial arrangements, including non-line-of-sight conditions. It is immune to ambient temperature, humidity changes and deliberate RF interference. No prior calibration is needed, and the architecture supports scalable parallel operation. Its integration of harmonic beamforming, broadband spectral probing and machine learning enables a compact and reliable platform for real-time liquid identification.

Future developments may focus on increasing the metasurface aperture to support narrower beams, and hence finer angular resolution, reducing the system's power consumption for battery-powered or embedded deployments, and incorporating online learning algorithms for continuous adaptation to new substances. Integration with portable or handheld platforms could further enhance usability in field conditions.

This work establishes a versatile and scalable approach to wireless chemical sensing, with broad potential across security screening, environmental monitoring and industrial inspection.
